# Different response of the taxonomic, phylogenetic and functional diversity of birds to forest fragmentation

**DOI:** 10.1038/s41598-020-76917-2

**Published:** 2020-11-23

**Authors:** Michał Bełcik, Magdalena Lenda, Tatsuya Amano, Piotr Skórka

**Affiliations:** 1 Institute of Nature Conservation, Polish Academy of Sciences, Mickiewicza 33, 31-120 Kraków, Poland; 2 School of Biological Sciences, University of Queensland, Brisbane, QLD 4072 Australia

**Keywords:** Biodiversity, Community ecology, Forest ecology

## Abstract

Habitat fragmentation is considered as major threat to biodiversity worldwide. Biodiversity can be described as taxonomic, functional and phylogenetic diversity. However, the effect of forest fragmentation on taxonomic, phylogenetic and functional diversity is barely understood. We compare the response of taxonomic (species richness), phylogenetic and functional diversity of birds to forest fragmentation. We hypothesised that with increasing forest patch isolation and/or decreasing patch size the diversity of birds decreases but only if certain thresholds of fragmentation metrics are reached. Specifically, we hypothesized that out of the three diversity components the taxonomic diversity is the most sensitive to forest fragmentation, which means that it starts declining at larger patch size and higher connectivity values than phylogenetic and functional diversity do. We compared the three biodiversity metrics of central European bird species in a large set of forest patches located in an agricultural landscape. General additive modeling and segmented regression were used in analyses. Habitat fragmentation differentially affected studied biodiversity metrics. Bird taxonomic diversity was the most responsive towards changes in fragmentation. We observed an increase in taxonomic diversity with increasing patch area, which then stabilized after reaching certain patch size. Functional diversity turned out to be the least responsive to the fragmentation metrics and forest stand characteristics. It decreased linearly with the decreasing isolation of forest patches. Apart from the habitat fragmentation, bird taxonomic diversity but not phylogenetic diversity was positively associated with forest stand age. The lower share of dominant tree species, the highest taxonomic diversity was. While preserving a whole spectrum of forests (in terms of age, fragmentation and size) is important from the biodiversity perspective, forest bird species might need large, intact, old-growth forests. Since the large and intact forest becomes scarcer, our study underscore their importance for the preservation of forest specialist species.

## Introduction

In the Anthropocene land use changes such as intensive agriculture and urbanization have led to habitat fragmentation and loss which are primary drivers of species extinctions worldwide^1–4^, however there is often disagreement to the extent to which fragmentation itself is to be blamed for the biodiversity loss^5^. The biodiversity decline may be initiated if the amount of available habitat falls below a certain, often species-specific, threshold level^6^. This may lead to the emergence of non-linear response of biodiversity to habitat fragmentation^7^.

Traditionally, taxonomic diversity (species richness) has been the most commonly used index of the biodiversity^8^. Phylogenetic diversity is another key component of biodiversity, reflecting life’s evolutionary heritage. Functional diversity is also an important feature of biological assemblages, having large impact on the rate and reliability of ecosystem processes^9,10^. There is often high redundancy in functional and phylogenetic diversity in species communities^11,12^, in which case species loss may have no effect on ecosystem processes. Continued species extinction however invariably leads to irreversible degradation of ecosystem functions^13^. Thus, the three above-mentioned biodiversity components may show different responses to measures of fragmentation.

Several studies investigated the impact of habitat fragmentation on taxonomic, phylogenetic and functional diversity metrics. Some authors indicate a lack of significant impact of fragmentation on phylogenetic diversity^14,15^, while other suggest that it might be affected by the edge effect and ecotone zones^16^. Functional diversity may be sensitive to a decrease in area and connectivity of habitat patches^17–20^. It is believed that fragmentation primarily affects specialist species and, to a lesser extent, generalists mostly via reduced connectivity^21,22^. However, those results may vary across different regions^23^ and specialist groups^24^.

Central European bird species, especially forest bird species, serve as an excellent group for understanding the effects of fragmentation on biological systems. They occur in landscapes highly affected by agriculture and urbanization, yet presenting different degrees of habitat fragmentation^25–28^. They also encompass a wide range of taxonomic functional and phylogenetic diversity^29^. Strong fragmentation favors generalists that are able to survive in smaller habitat patches than specialists^30,31^. In Europe, a decline of diversity of both farmland and woodland species is observed^32–34^. This decrease is more pronounced in species inhabiting farmlands than in species inhabiting forests^35^ mostly because farmland is constantly changing^32^ and is more prone to climate change. However, intensive forestry including salvage logging puts at risk forest birds, especially in Poland, where apparent conflict between government, foresters and conservationists have arisen in recent years^36,37^.

Evidence from studies on bird assemblages suggests that forest size and isolation have negative effect on taxonomic diversity^38^, functional diversity^18^, and phylogenetic and functional diversity combined^39^. Moreover, those effects vary markedly between generalist and specialist species^40^. However, there have been only a few studies focusing on comparing the responses of different biodiversity components to changes in patch size and isolation in one complex study.

The aim of our study is to compare the response of taxonomic, phylogenetic and functional diversity of birds to forest fragmentation metrics. We have decided to study the response of all of the bird species found within those forest patches and forest specialist only. Following hypotheses were tested:

### Hypothesis 1

With increasing patch isolation and/or decreasing patch size the diversity of birds decreases but only if certain thresholds of fragmentation metrics are reached. We expected that the diversity of forest specialists should be more sensitive to forest fragmentation (decrease faster) than the diversity of all of the bird species, since the latter also includes some farmland and ecotone species that may respond positively to fragmentation.

### Hypothesis 2

Out of the three diversity components taxonomic diversity is the most sensitive to forest fragmentation, which means that it starts declining at larger patch size and higher connectivity values than phylogenetic and functional diversity do. We have formulated that hypothesis both for forest specialists and all of the bird species.

We expected this because there is often high redundancy in phylogeny and function in species assemblages. Moreover, we expected that phylogenetic diversity drops at larger patch size and higher connectivity values than functional diversity because there may be convergence in traits among phylogenetically-distant species and thus function in ecosystems.

## Materials and methods

### Study area

**Figure 1:**
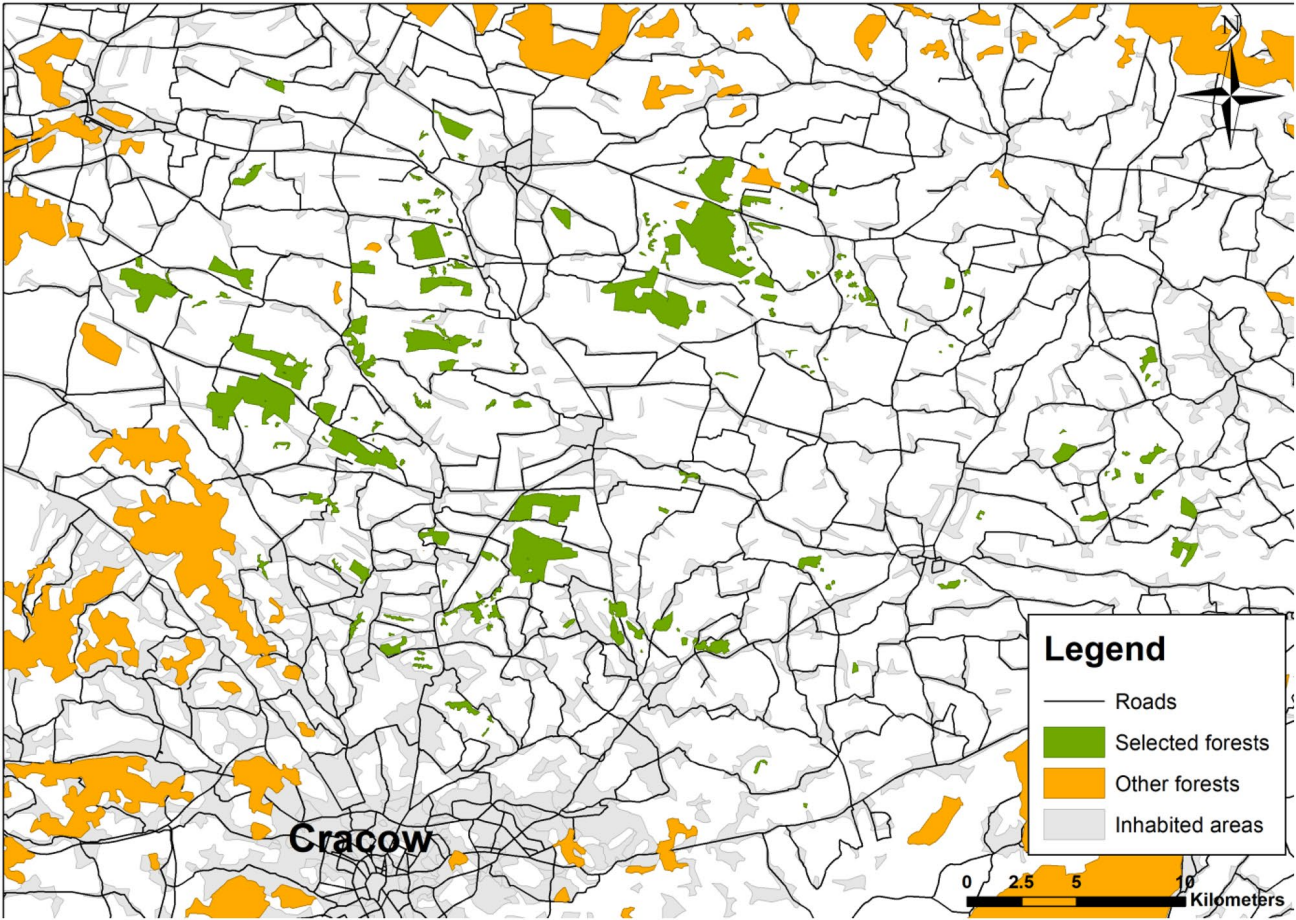
Map of the study area, with study forest patches marked in green, and other forests marked in orange. Created by Michał Bełcik using ArcMap 10.1.

The study has been conducted in the southern part of Poland, in the province of Małopolska, in an area encompassing 1097 square kilometres north of Cracow. We have chosen 163 forest patches located in an agricultural landscape (Fig. 1). Those were mostly mixed stands, both managed by the Polish State Forests Holding and private entities (supervised by the former entity). All these forest patches were habitat islands (not part of a larger continuous forest complex) and differed in size and isolation.

### Forest characteristics

For each forest patch, we collected a range of parameters to best capture the key characteristics of a forest stand which could possibly be important for local bird species (Table 1). They were measured and averaged for every single patch. Also, we used Forest Data Bank (www.bdl.lasy.gov.pl) as a data source for some of the forest patches. Where that data was not available, we have calculated those parameters in accordance to the guidelines of Forest Bureau for Forest Management and Geodesy^41^. Three the most commonly studied metrics of habitat fragmentation: patch size and two proximity indices were measures of forest fragmentation of primary interest. The isolation metrics were nearest neighbour distance (NND) and proximity index (PROX). Those were calculated using the Patch Analyst toolbox of the ArcGis ver. 10.1, which uses the same method to calculate landscape metrics as Fragstats software^42^. To avoid confounding effects, patch size and isolation metrics were selected in a way the correlation coefficients among them were low and non-significant (all values of the coefficient were below 0.2).

**Table 1:** Stand parameters and isolation metrics of studied forest patches.

Parameter	Type of parameter	Description	Log-transformed	Range	Mean ± SD
Forest area	Fragmentation variable	Total area of forest patch (in hectares)	Yes	0.38–582.33	37.28** ± **89.52
Forest age	Stand parameter	Mean age of dominant tree species in main stand storey (in years)	No	10–112	58.18** ± **24.30
Share of dominant species	Stand parameter	Expressed on the integer scale of 0–10 (with 10 being the highest result), the share of dominant tree species in main stand storey	No	2–10	–
Stand density	Stand parameter	Mean density of forest stand (representing percentage of forest bottom shaded by the tree canopy) (in %)	No	30–100	66.13** ± **15.80
Coniferous species	Stand parameter	Percentage of coniferous species in main stand storey (in %)	No	0.00–100.00	21.01** ± **26.04
Shape index (SI)	Fragmentation variable	Shape Index of forest stand	Yes	1.110–3.528	1.790** ± **0.504
Nearest neighbour distance (NND)	Fragmentation variable	Shortest straight-line distance between a focal patch and its nearest neighbour (in m)	Yes	16.53–3509.19	269.26** ± **701.36
Proximity index (PROX)	Fragmentation variable	Sum, over all patches whose edges are within the 2.5 km radius of the focal patch, of each patch size divided by the square of its distance from the focal patch	Yes	0.00–1845.83	78.86** ± **251.92

### Bird observations

Field surveys were carried out between the 1st of April and 31st of May 2017 by a team of three experienced birdwatchers. Each of those observers had the assigned set of forest patches Each forest was visited three times. We have divided that period into three 20-days rounds (1–20 April, 21 April–10 May, 11–31 May). In each of those periods, forest patches were surveyed once. Surveys started at around 5 a.m. and usually lasted till 11 a.m. During surveys an observer noted the starting time, then moved through forest in a random direction, trying to cover as much of the forest patch as possible. Each observer noted all species and the exact time of the first observation for each species heard or seen within a patch. From survey starting time in forest and time of observation of the first individual a species abundance index within a patch was estimated adopting the Michaelis-Mentien model^43^ (Skórka et al. in prep). The survey ended if none new species was recorded for ten minutes (Skórka et al. in prep.). We have decided not to utilize a survey that assumes spending fixed time on every site since our forest patches varied markedly in size. We have instead decided to include both the effects of time and space in our models, which has been shown to increase the modelling accuracy^44^.

We have divided our bird observations into two groups, for which we performed our analysis. The first was all of the bird species observed in those forest patches, including both forest specialists and generalist (further in this text—“all species”). The second group was a subset of forest specialists^45^, which we created in accordance with the PanEuropean Common Bird Monitoring Scheme (www.ebcc.info). We refer to that group in this manuscript as “forest specialists”.

### Phylogenetic and functional diversity indices

As bird biodiversity metrics (for both all of the bird species and forest specialists) within each patch we computed: taxonomic diversity, phylogenetic diversity and functional diversity. Each of those metrics were computed for the alpha diversity level^46^. For measuring the phylogenetic diversity, we used the mean nearest taxon distance—MNTD^47^. This metric averages the subset of the possible pairwise distances extracted from a phylogenetic tree, where only the shortest distances between taxa are considered^47^. Phylogenetic tree was obtained from the BirdTree project website^48^ (www.birdtree.org).

For measuring the functional diversity, we used the functional richness—Fric^10^. This measure quantifies the amount of a niche space occupied by the species within a community. This measure was chosen since it is independent of abundance, thus a section of niche space is considered to be occupied even if only low abundance occurs within it. This characteristic of this measure enabled us to fully capture the functional diversity of small forests and better study the possible effects of fragmentation on this measure. This metric was also chosen because it turned out to be the most sensitive to forest patch size and isolation as compared to other metrics (e.g. functional dispersion, functional evenness and functional divergence), as described by Mason et al.^10^. For calculating diversity measures, we used “picante”^50^ and “FD” packages^51^ in R. We used species traits linked with functioning of forest ecosystems (Table S1). These traits were related to diet, reproductive mode, lifespan, migratory behaviour, social behaviour. All these traits are linked e.g. with species interactions, nutrient cycling, seed dispersal, using space, thus have impact on forest ecosystem functioning.

### Data preparation and analysis

All statistical analyses were performed in R statistical software^52,53^. The first step in our analysis was to test which patch characteristics and isolation metrics can be used as explanatory variables in modelling bird diversity in forest patches. For that, we have used the “mgcv” package^54^. We constructed a general additive model for each of the response variables: taxonomic diversity, functional richness, and phylogenetic diversity for bird assemblages including (1) all species and (2) only forest specialists. Models included all of the explanatory variables that we considered might be explaining that diversity variability (Table 1). The variance inflation factor was equal to 1.43 for the percentage of coniferous species, and below that value for other explanatory variables. Variables represented two groups – those that described patch size and isolation (fragmentation variables) and those that described the stand parameters potentially related to the quality of forest patches. For mean forest age and stand density a linear relationship was assumed, but for most, we have assumed a non-linear relationship between explanatory variables and response variables to identify threshold values We also included the interaction between geographic coordinates modeled as smoothed function for all models to control for spatial autocorrelation and abiotic heterogeneity^55^, and the number of species as a covariate for models with functional diversity as the response variable, due to usually strong positive association between the number of species and functional diversity. Variables representing fragmentation indices were logarithmically transformed to avoid impact of detached observations (Table 1). To validate our models, we used a gam.check() function from the “mgcv” package^54^, which produces diagnostic information, along with four residual plots. This function produces some diagnostic information about the fitting procedure and results, including a check whether the basis dimension for a smooth is adequate (not too low), along with four standard diagnostic plots. Our results showed that we had used a similar basis dimension (i.e. number of k-values) for our model as suggested, and plots produced showed a general good fit of the models. We also used the concurvity() function from the same package, which produces summary measures of concurvity between model components. All these checks revealed that the models were correctly constructed.

The second step in data analysis was to identify the response thresholds of diversity metrics to forest patch size and isolation with segmented regression. We calculated thresholds for each explanatory variable that showed a non-linear association with biodiversity metrics, using the lm.br() function from the “lm.br” package^56^. This function performs a significance tests for a changepoint in linear or multiple linear regression, and computes confidence intervals and confidence regions with exact coverage probabilities for the changepoint.

## Results

### Bird responses to fragmentation metrics

In total, 94 bird species were observed, of which 44 were forest bird species. The mean number of species per one survey at the given forest patch was 25 (SE = 7, min = 4, max = 42).

**Figure 2:**
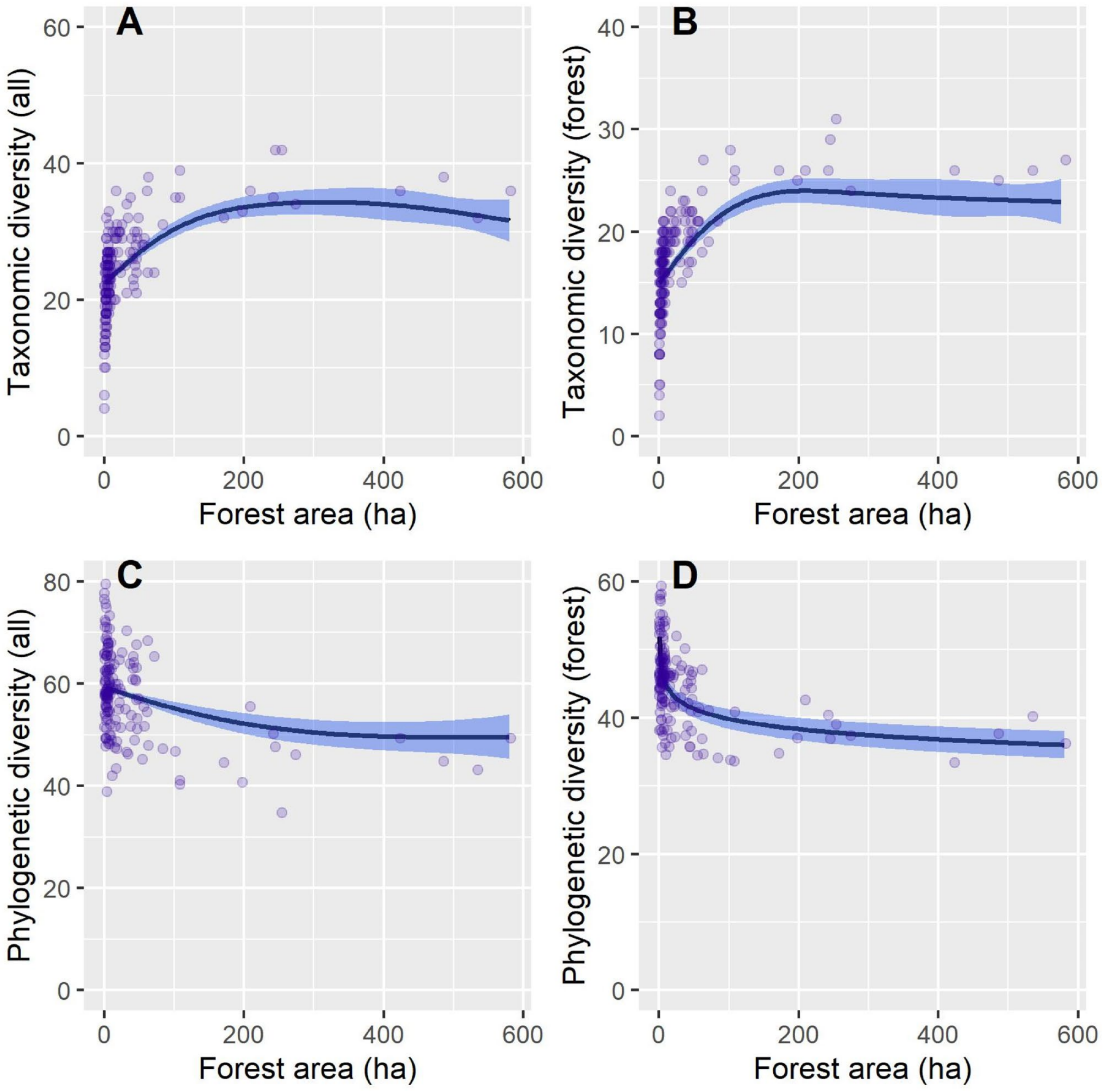
Response curves, derived from General Additive Modelling, showing the relationship between taxonomic and phylogenetic diversity (calculated for all of the bird species and forest specialist group) and forest patch area (in hectares).

**Figure 3:**
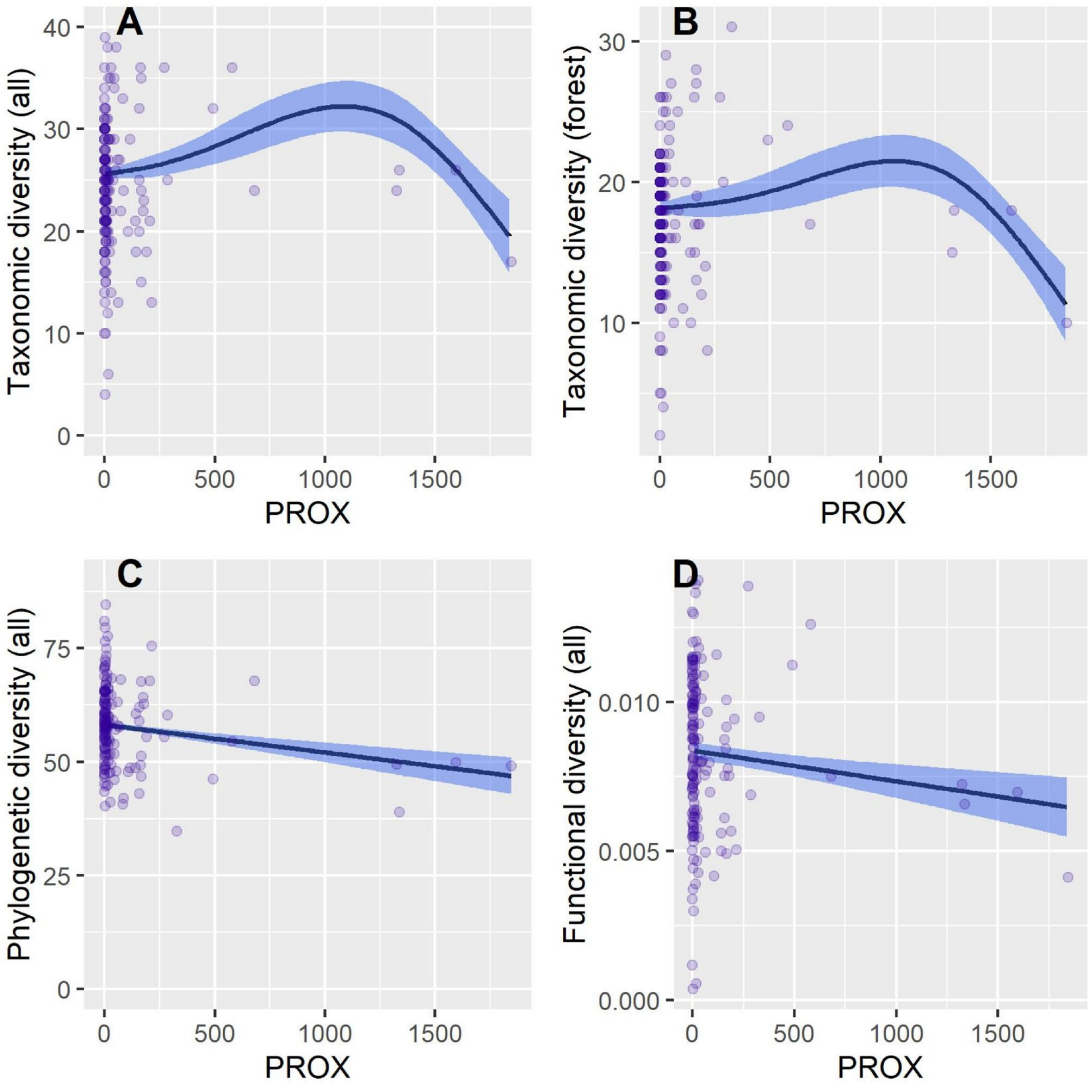
Response curves, derived from General Additive Modelling, showing the relationship between different metrics of bird diversity (for all of the study species and forest specialist group) and the amount of forest patches calculated for 2-km buffer (PROX).

Results of general additive models showed varying biodiversity metrics responses to isolation metrics and stand parameters (Table 2). The area of a forest patch, proximity index and forest age had significant influences on diversity metrics. The taxonomic diversity (all species and forest specialists) increased non-linearly with the forest area (Fig. 2A,B). However, phylogenetic diversity decreased non-linearly with forest area and this decrease was rapid at low forest sizes (Fig. 2C,D). Functional diversity did not respond to the forest patch area. Taxonomic diversity of all species and forest specialist was highest at high (a low value of proximity index) and moderate habitat isolation (Fig. 3A,B). Phylogenetic and functional diversity indices for all bird species decreased linearly with decreasing habitat isolation (increasing values of proximity index, Fig. 3C,D). However, phylogenetic and functional diversity of forest specialists did not respond to this forest isolation index (Table 2). Another isolation metric, the nearest neighbor distance had a significant positive association with taxonomic diversity of all birds (Table 2, Fig. S1A).

**Table 2:** The effect of environmental variables on bird diversity components at patch characteristics and isolation metrics.

Explanatory variables	Response variables					
	Species richness of all birds	Functional diversity of all birds	Phylogenetic diversity of all birds	Species richness of forest bird specialists	Functional diversity of forest bird specialists	Phylogenetic diversity of forest bird specialists
**GAM estimates of function slopes with standard errors (in brackets) for explanatory variables with assumed linear response**						
Intercept	**19.06994** (2.051163)***	**0.00596** (0.00085)***	**63.23644** (3.35101)***	**11.92428** (1.44099)***	**0.07706** (0.01098)***	**47.20254** (3.04480)***
Forest age	**0.09464** (0.01640)***	**0.00001** (**0.00001) ’**	**− 0.09458** (**0.02574)** ***	**0.06845** (**0.01137)** ***	0.00002 (0.00009)	**− **0.01585 (0.02487)
Stand density	0.00084 (0.02351)	**0.00002** (0.00001)*	0.00534 (0.03829)	0.02145 (0.01646)	0.00007 (0.00012)	**− **0.01375 (0.03414)
**Explanatory variables included as splines to control for potential non-linearity, with degrees of freedom presented**						
NND	**Df = 1.000’**	Df = 1.000	Df = 1.000	Df = 3.033	Df = 1.000	Df = 1.000
PROX	Df = 2.986*	**Df = 1.000’**	Df = 1.000**	Df = 3.073*	Df = 1.000	Df = 1.000
Forest area	Df = 2.908***	Df = 1.000	Df = 1.801**	Df = 3.225***	Df = 1.903	Df = 1.000***
Number of species	Not included	Df = 1.845***	Not included	Not included	Df = 1.000***	Not included
Coniferous species	Df = 1.496*	Df = 1.027	Df = 1.000	Df = 1.000***	Df = 2.552**	Df = 1.003
Share of dominant species	Df = 1.000*	Df = 2.401	Df = 1.000	Df = 1.000*	Df = 2.021	Df = 1.000
SI	Df = 1.000***	Df = 1.000	Df = 1.000	Df = 1.000**	Df = 1.908*	Df = 1.000

Statistically significant effects are emboldened: ***P < 0.001, **P < 0.01, *P < 0.05, ‘P < 0.10.

### Responses to forest parameters

Forest stand characteristic was another group of factors that influenced different components of bird diversity (Table 2). Forest age was positively associated with the species and functional diversity but negatively with the phylogenetic diversity of all bird species. Similar findings were found for taxonomic diversity of forest specialists (Table 2, Fig. S2B). Stand density turned out to have a positive association with the functional diversity of all bird species. Percentage of coniferous species had positive correlation with the taxonomic diversity of all birds and forest specialists, as well as positive non-linear association with the phylogenetic diversity of forest specialists (Table 2, Fig. S3A,B). Share of dominant species was negatively associated with taxonomic diversity of all birds and forest specialists and this relationship was linear in both cases (Table 2, Fig. S4A,B).

### Threshold responses

Analysis of thresholds in the response of birds to fragmentation metrics and forest characteristics showed statistically significant changepoints for different bird biodiversity components (Table 3). Taxonomic diversity was the least sensitive metric to forest fragmentation and forest stand characteristics (Table 3). The threshold response of the phylogenetic diversity to forest patch size was different than expected, i.e. phylogenetic diversity decreased very quickly with increasing forest patch size but then stabilized at low diversity values and large forest patch size. There were no threshold responses of functional diversity to fragmentation metrics but there was a threshold response for a share of coniferous trees (Table 3).

**Table 3:** The response thresholds of diversity metrics to forest patch size and isolation.

Response variable	Explanatory variable	Changepoint	Significance level
Species richness of all bird species	Proximity Index (PROX)	570.005	0.185
	Forest patch area	**6.038**	< 0.001***
	Percentage of coniferous species	**68.835**	< 0.001***
Species richness of forest specialists	Proximity Index (PROX)	**451.550**	**0.051‘**
	Forest patch area	**10.426**	< 0.001***
Phylogenetic diversity of all bird species	Forest patch area	**123.518**	0.002**
Functional diversity of forest specialists	Percentage of coniferous species	**70.066**	< 0.001***
	Shape Index (SI) of forest patch	2.474	0.452

Thresholds were calculated only for variables that had statistically significant effect on diversity measures.

Statistically significant effects are emboldened: ***P < 0.001, **P < 0.01, *P < 0.05, ‘P < 0.10.

## Discussion

In our study we showed that habitat fragmentation affected the studied biodiversity components differently.. Taxonomic diversity was most sensitive to changes in fragmentation indices and forest parameters, compared to the functional and phylogenetic diversity (Table 2). We also observed that taxonomic diversity of all birds was susceptible to changes in a wider variety of forest parameters than taxonomic diversity of forest specialists. We suggest that it might be due to the fact that generalists are usually more taxonomically diverse and have wider ecological preferences than specialists^57^. However, against our previous assumption, it turned out that taxonomic diversity drop at lower patch size than phylogenetic diversity (Table 3). That effect was observed for all of the bird species and forest specialists as well. We were unable to verify the same assumption for the connectivity indices. Functional diversity, however, turned out to be the least responsive to the forest patch size and isolation metrics, as well as forest stand characteristics. That goes in line with our initial hypothesis that there is a significant redundancy of functions within bird assemblages. Moreover, according to Cadotte et al.^58^, functional diversity is the least susceptible to the changes in forest cover and deforestation processes.

There are several possible explanations of the observed pattern of response to fragmentation metrics. Previous studies have shown a positive relationship between habitat patch area and taxonomic diversity^3,30,59^. In our study, we saw an increase in taxonomic diversity across both bird groups with increasing patch area, which then stabilized after reaching a certain patch size (Fig. 2A,B). It is interesting to note that beyond this threshold, an increase in patch area does not yield an increase in taxonomic nor phylogenetic diversity. This would suggest that large, undisrupted interior areas are vital to maintaining the diversity of both forest specialists and all of the bird taxa^60,61^. However, we did not observe habitat loss driving changes in phenotypic traits (i.e., no significant relationship between patch size and functional diversity was found, Table 2) as it was also reported elsewhere^11,38,59^. A possible explanation is that in our study area, even a relatively small forest patch was enough to harbor a range of niches^57,62^, that could maintain a functionally diverse avian population. It could also indicate a high functional redundancy within bird communities^58^.

The same process could explain a negative, linear relationship between the PROX and functional diversity of all bird species (Fig. 3D). However, this negative relationship could also be explained by the positive influence of fragmentation on biodiversity^5^. Furthermore, results for the phylogenetic diversity of all bird species (Fig. 3C) could indicate a rather opposite explanation^63^. For the taxonomic diversity (for both analyzed groups), we can see a non-linear relationship with proximity index (Table 2, Fig. 3A,B). The shape of the curve might indicate the influence of environmental gradients, dictated by the spatial composition and distance between the forest patches^64,65^. Evidence from other studies indicates that gradients of habitat cover can result in high taxonomic diversity at intermediate fragmentation level^30,66,67^. Nearest neighbour distance had a linear positive influence on taxonomic diversity of all of the bird species. That could once again support the hypothesis of the positive influence of habitat fragmentation on taxonomic diversity^5^.

The least important fragmentation metrics were shape index (SI) and NND (Table 2, Fig. S1). SI had a significant, positive linear influence on taxonomic diversity (for both groups) and a non-linear positive influence on the functional diversity of forest specialists. A possible explanation of this result could point to studies that indicated a high taxonomic diversity in the forest-field ecotone^60,68^. Higher SI means a longer, more complex border between a forest and field habitat, which generates a greater area of ecotone zones. Such zones are a highly heterogeneous environment (both in terms of structure and habitat composition), able to sustain a greater diversity of birds species^60^.

### Threshold responses

Prior to the analysis, we had expected the existence of forest patch area threshold for biodiversity of both all of the bird species and forest specialists^69,70^. We have also expected an existence of such threshold for forest canopy density^71^ and forest age^72^, especially for forest specialist species. Our analysis showed that patch size and isolation thresholds do exist. The most important thresholds describe the relationship between forest patch area and taxonomic diversity (of both studied bird subsets) and phylogenetic diversity of all bird species. It confirms our initial hypothesis that after the decrease to a certain patch size, taxonomic diversity metric will start to drop significantly. It also supports evidence from other studies showing that habitat specialists may be more severely impacted by habitat fragmentation than generalists^73,74^. It has a number of practical implications, because it shows that in order to preserve biodiversity of bird assemblages of mixed rural landscape (as it is a goal of many European conservation programmes), it is vital to ensure that certain size of patches must be maintained^75,76^. It is important to note that this threshold was definitely highest for phylogenetic diversity, and differs markedly between diversity metrics. That indicates phylogenetic susceptibility to fragmentation and underlines the need to take all of the diversity measures into account when designing efficient conservation plans.

The percentage of coniferous species, below which functional diversity of forest specialists started to decline, was about 70% (Table 3). This metric reflects homogeneity of the stand –the higher the amount of coniferous species is, the more homogeneous forest patch is. This underlines the need for maintaining diverse stands that would include a certain proportion of coniferous species in devising forest management strategies^60^. In spite of our initial assumptions, we found no evidence of existence of significant forest patch age or stand density thresholds on forest specialists. That may be due to the fact that most of the forest patches studied were of medium age and moderate canopy density, which are not usually characteristics of an old-growth forests that are favoured by a large proportions of woodland species.

Our results underscore the importance of considering the thresholds together with biodiversity metrics, because these measures may be differently related to the habitat fragmentation. So far, many studies suggest that the effect of habitat fragmentation on extinction thresholds to be as likely positive as negative^69,77–79^. Because biodiversity metrics differ in their response to changes in habitat features, a one process could trigger different kinds of responses between biodiversity metrics. For example, a taxonomic diversity may increase after exceeding a certain threshold of habitat patch area, and phylogenetic diversity may flatten after reaching similar threshold. It would indicate that between these two thresholds there is an optimum in which a high number of species and a large phylogenetic diversity persists.

### Responses of other variables

Among other variables that were shown to have an influence on bird diversity metrics, the most important one was the age of forest stand. It had a positive influence on taxonomic, phylogenetic and functional diversity of all bird species, and on a taxonomic diversity of forest specialists. This is in line with the findings of other studies focusing on bird diversity in forest habitats^60^. Response curve was slightly steeper in case of the taxonomic diversity of all bird species, than in case of the taxonomic diversity of forest specialists (Fig. S2A,B). That would indicate that the older the tree stand is, the more specialized the forest bird species are in that stand. It is also important to note that the slope coefficient of the taxonomic diversity of all species was the highest among all three types of diversity. That leads to the conclusion that it is most susceptible to the changes in forest stand age caused by, for example, clearcuts and timber production.

According to classical niche-assembly models, the abundance and occurrence of species within communities are determined, among other factors, by the diversity of resources and habitats available^80^. The results from our study show that the most diverse forests were those with a high percentage of coniferous species in the main forest stand (Fig. S3) and with a low share of dominant species in forest stand (Fig. S4). This indicates the preference towards mixed, multi-species stands, which has already been signalled in previous studies^81,82^.

It is noteworthy, however, that under different latitude, studies like our could yield potentially different results. Tropic ecosystems have higher diversity metrics and more complex biotic interactions among species than ones we see in temperate zones, which in turns leads to a wider variety of ecosystem functions in a given fauna pool. Therefore, reduction of some functional groups caused by fragmentation can be more apparent in tropical ecosystems. This could also create different thresholds of diversity metrics in tropics than in temperate zones. However, some similarities could also be observed. For example, studies have shown that disturbance caused by fragmentation also favours generalist species in tropics^83^. Possible frontiers for further studies stemming from our research could include a more complex analyses in which bird species would be grouped by their functional characteristics (e.g. foraging behaviour, nesting substrate). Calculating functional diversity metrics within such functional groups could reveal a new findings and show which functions are affected by the fragmentation the most.

## Conclusions

Forest fragmentation is not universally negative on every aspect of bird communities diversity. It probably generates a high density of environmental gradients, which might be one of the most important drivers of diversity in community composition^65–67^. We believe that high habitat diversity of rural landscape caused by habitat fragmentation might be a positive feature for biodiversity provided that the size of the forest patches does not fall below certain area thresholds^61,84–86^.

The differences of responses between biodiversity measures of either all bird species or forest specialists is more significant than the difference in responses of one diversity measures between all bird species and forest specialists. When we compare the responses of taxonomic diversity and shared evolutionary (both for all species and forest specialist) history to patch area, we see that the pattern of response is similar for both groups, and the response between taxonomic and phylogenetic diversity is non-consistent. The same could be observed for a number of other variables, like proximity index or forest stand age.

Understanding impact of habitat fragmentation on biological systems requires analyses that include various diversity components. We believe that a broader, more complex approach towards biodiversity is also necessary while studying natural (like population dynamics) or anthropogenic processes (like habitat fragmentation or invasion of alien species^87,88^). Focusing only on one diversity metric might lead to inaccurate conclusions since different metrics might respond in a different way to the same studied variable, as we have proven in case of proximity index. Our results also indicate that devising conservation strategies for various groups of birds is a multi-faceted dilemma, which should be important in decision making, at least in temperate zone. From the point of view of biodiversity as a whole, it may be important to maintain the full spectrum of forests that would represent different age, area, isolation or stand parameters. Considering this perspective, forest fragmentation might not necessarily be considered as a negative phenomenon, as it probably increases the density of ecotonic zones and thus the heterogeneity of the environment (both in terms of structure and habitat composition). This may allow for the penetration of typical farmland species into the forest patches, thus increasing the biodiversity in the given patch. From the point of view of forest specialists, however, it is necessary to preserve large and compact forests, consisting of old trees of different species composition and a dense canopy. Considering the scarcity of such large forests, our results underscore the importance of protecting those few that are still remaining across a lowland landscape of Central Europe.

## Supplementary information

Supplementary Information

Supplementary Information

Supplementary Information

Supplementary Information

Supplementary Information
