# Supplementary material for: Different response of the taxonomic, phylogenetic and functional diversity of birds to forest fragmentation
Source: Sci Rep. 2020 Nov 23;10:20320. doi: 10.1038/s41598-020-76917-2 (PMC7683534; doi:10.1038/s41598-020-76917-2)
Supplement: Supplementary file 1 — Supplementary Information [file 41598_2020_76917_MOESM1_ESM.pdf]

Different response of the taxonomic, phylogenetic and functional diversity of birds to forest fragmentation

Michał Bełcik[1,*], Magdalena Lenda[1,2], Tatsuya Amano[2], Piotr Skórka[1]

[1] Institute of Nature Conservation, Polish Academy of Sciences, Mickiewicza 33, Kraków 31-120, Poland. ORCiD ID: https://orcid.org/0000-0001-8901-2064

[2] University of Queensland, School of Biological Sciences, Brisbane QLD 4072, Australia.

[*] Correspondence should be addressed to e-mail: belcik@iop.krakow.pl

Table S1. Functional traits used for calculating functional diversity metrics

Variable	Description	Units
LengthU_MEAN	Mean length of the bird, unsexed	cm
WingU_MEAN	Mean length of the wing, unsexed	mm
WingM_MEAN	Mean length of the wing, male	mm
WingF_MEAN	Mean length of the wing, female	mm
TailU_MEAN	Mean length of the tail, unsexed	mm
TailM_MEAN	Mean length of the tail, male	mm
TailF_MEAN	Mean length of the tail, female	mm
BillU_MEAN	Mean length of the bill, unsexed	mm
BillM_MEAN	Mean length of the bill, male	mm
BillF_MEAN	Mean length of the bill, female	mm
TarsusU_MEAN	Mean length of tarsus, unsexed	mm
TarsusM_MEAN	Mean length of tarsus, male	mm
TarsusF_MEAN	Mean length of tarsus, female	mm
WeightU_MEAN	Mean weight in breeding season, unsexed	grams
WeightM_MEAN	Mean weight in breeding season, male	grams
WeightF_MEAN	Mean weight in breeding season, female	grams
Sexual dimorphism	Difference between male and female in size and plumage colour	1: yes; 0: no
Clutch_MIN	Minimum clutch size	number of eggs

Clutch_MAX	Maximum clutch size	number of eggs
Clutch_MEAN	Mean clutch size	number of eggs
Broods per year	Mean number of broods per breeding season (replaced broods are not included)	number of broods
EggL_MEAN	Mean length of the egg	mm
EggW_MEAN	Mean width of the egg	mm
Egg mass	Mean weight of the egg	grams
Young	Type of young	AL = altricial, SA = semi-altricial, PR = precocial
Association during nesting	Association of adults during nesting	S = solitary, SC = semi-colonial, C = colonial
Nest type	Type of nest	G = ground, on ground directly; H = hole, in tree, bank, ground, crevice; OA = open-arboreal, cup in bush, tree, on cliff ledge; CA = closed-arboreal; GC = ground close, nest in tussock very close to ground but not directly on ground, hidden in surrounded vegetation
Nest building	Sex building the nest	M = male, F = female, B = both, N = none
Mating system	Type of mating system	M = monogamous, PG = polygynous, PA = polyandrous, PM = promiscuous
Incubation period	Mean length of eggs´ incubation	days
Incubation sex	Sex incubating eggs	M = male, F = female, B = both, N = none
Hatching	Type of hatching	AS = asynchronous young hatch within 2 days or more, SY = synchronous, young hatch within 1 day
Eggshells	Eggshells are left in the nest or not	1: yes; 0: no
Nestling period	Average age of young when leaving nest	days
Fledging period	Average age of young when fledging	days
Parental feeding	Average age when young are not regularly fed by parents	NA = birds which feed young but the period is unknown, 0 = birds which do not feed young, days
Age of independence	- Average age when young totally separate off parents - young are not fed or protected by parents	days
Feeding independence	Average age when young are independent of feeding by	days

	parents, calculated as mean from Parental feeding and Age of independence	
Age of first breeding	Average age of the first breeding	years
Life span	Maximum life span recorded in wild	years
Post-fledging mortality	Mean mortality of young in the first year of their life	%
Mortality of adults	Mean annual mortality of adults	%
Association outside the breeding season	Association of adults outside breeding season	GR = gregarious, PA = in pairs, SO = solitary
Territoriality	Defence of a territory (defended area occupied exclusively by a single bird, pair or larger social unit)	1: yes; 0: no
Sedentary	Species lives in the same area in both breeding and non-breeding season	1: yes; 0: no
Facultative migrant	Species makes irregular shifts in non-breeding season	1: yes; 0: no
Short distance migrant	Species migrates within the range of the Western Palearctic in non-breeding season	1: yes; 0: no
Long distance migrant	Species migrates beyond the range of the Western Palearctic in non- breeding season	1: yes; 0: no
Deciduous forest	Species occupies deciduous forest in breeding area	1: yes; 0: no
Coniferous forest	Species occupies coniferous forest in breeding area	1: yes; 0: no
Woodland	Species occupies woodland, i.e. habitat with disperse vegetation, edge of forest, etc. with presence of full-grown trees in breeding area	1: yes; 0: no
Shrub	Species occupies shrub. i.e. habitat with disperse vegetation, bush, shrub, scrub, etc. without presence of full-grown trees in breeding area	1: yes; 0: no
Savanna	Species occupies savanna in breeding area	1: yes; 0: no
Tundra	Species occupies tundra in breeding area	1: yes; 0: no
Grassland	Species occupies grassland (lowland meadows and fields) in breeding area	1: yes; 0: no

Mountain meadows	Species occupies mountain meadows in breeding area	1: yes; 0: no
Reed	Species occupies swamps with reed in breeding area	1: yes; 0: no
Swamps	Species occupies swamps without reed in breeding area	1: yes; 0: no
Desert	Species occupies desert and semi-desert habitats in breeding area	1: yes; 0: no
Freshwater	Species occupies static and flowing freshwaters in breeding area	1: yes; 0: no
Marine	Species occupies marine habitats in breeding area	1: yes; 0: no
Rocks	Species occupies rocks (stony habitats, cliffs, crags etc.) in breeding area	1: yes; 0: no
Human settlements	Species occupies human settlements in breeding area	1: yes; 0: no
Folivore_Y	At least 10% of diet throughout the year composed of grass, leaves, small plants etc.	1: yes; 0: no
Frugivore_Y	At least 10% of diet throughout the year composed of fruits	1: yes; 0: no
Granivore_Y	At least 10% of diet throughout the year composed of grains, seeds and nuts	1: yes; 0: no
Arthropods_Y	At least 10% of diet throughout the year composed of arthropod	1: yes; 0: no
Other invertebrates_Y	At least 10% of diet throughout the year composed of invertebrates excepting arthropods	1: yes; 0: no
Fish_Y	At least 10% of diet throughout the year composed of fish	1: yes; 0: no
Other vertebrates_Y	At least 10% of diet throughout the year composed of vertebrates excepting fish	1: yes; 0: no
Carrion_Y	At least 10% of diet throughout the year composed of carrion	1: yes; 0: no
Omnivore_Y	Diet throughout the year composed of similar amount of plants and animals	1: yes; 0: no
Folivore_B	At least 10% of diet	1: yes; 0: no

	throughout the breeding season composed of grass, leaves, small plants etc.	
Frugivore_B	At least 10% of diet throughout the breeding season composed of fruits	1: yes; 0: no
Granivore_B	At least 10% of diet throughout the breeding season composed of grains, seeds and nuts	1: yes; 0: no
Arthropods_B	At least 10% of diet throughout the breeding season composed of arthropods	1: yes; 0: no
Other invertebrates_B	At least 10% of diet throughout the breeding season composed of invertebrates excepting arthropods	1: yes; 0: no
Fish_B	At least 10% of diet throughout the breeding season composed of fish	1: yes; 0: no
Other vertebrates_B	At least 10% of diet throughout the breeding season composed of vertebrates excepting fish	1: yes; 0: no
Carrion_B	At least 10% of diet throughout the breeding season composed of carrion	1: yes; 0: no
Omnivore_B	Diet throughout the breeding season composed of similar amount of plants and animals	1: yes; 0: no
