# Supplementary material for: Different response of the taxonomic, phylogenetic and functional diversity of birds to forest fragmentation
Source: Sci Rep. 2020 Nov 23;10:20320. doi: 10.1038/s41598-020-76917-2 (PMC7683534; doi:10.1038/s41598-020-76917-2)
Supplement: Supplementary file 2 — Supplementary Information [file 41598_2020_76917_MOESM2_ESM.pdf]

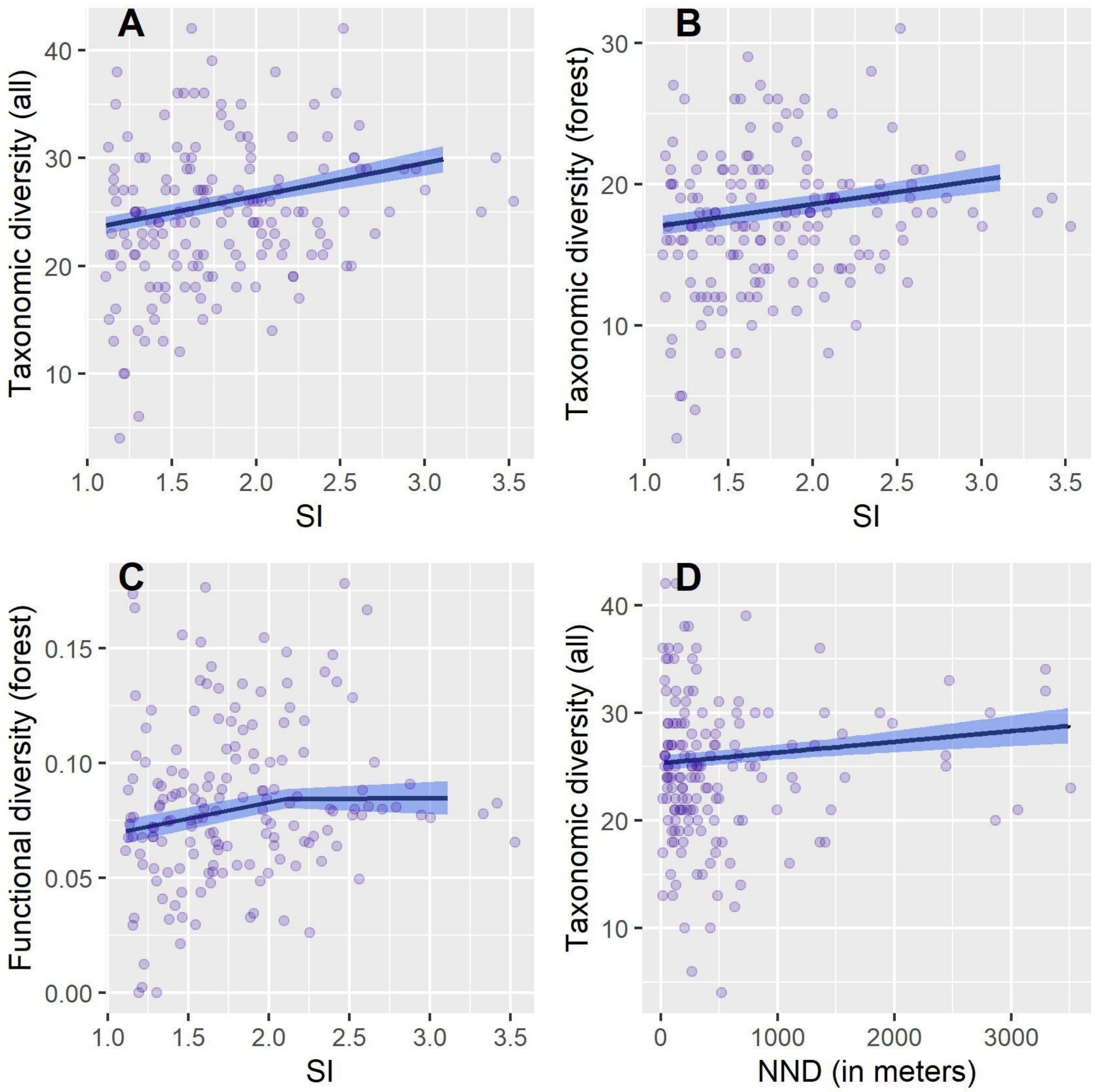

Figure S1. Response curves, derived from General Additive Modelling, showing the relationship between taxonomic and functional diversity (calculated for all of the bird species and forest specialist group) and forest patch shape index (SI) and nearest neighbour distance (NND).
