# Supplementary material for: Different response of the taxonomic, phylogenetic and functional diversity of birds to forest fragmentation
Source: Sci Rep. 2020 Nov 23;10:20320. doi: 10.1038/s41598-020-76917-2 (PMC7683534; doi:10.1038/s41598-020-76917-2)
Supplement: Supplementary file 3 — Supplementary Information [file 41598_2020_76917_MOESM3_ESM.pdf]

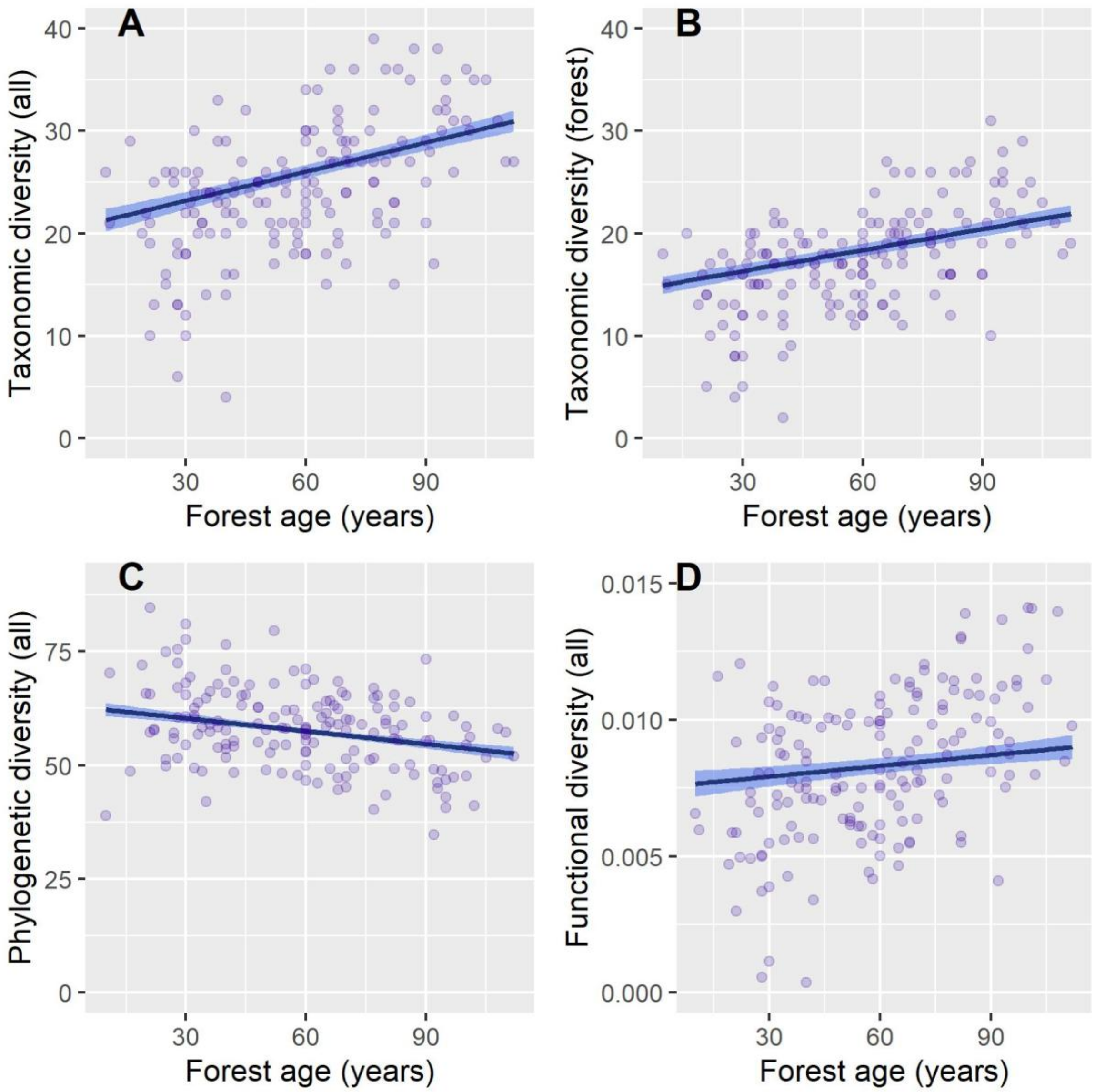

Figure S2. Response curves, derived from General Additive Modelling, showing the relationship between different metrics of bird diversity (for all of the study species and forest specialist group) and age of the forest patches.
