# Supplementary material for: Different response of the taxonomic, phylogenetic and functional diversity of birds to forest fragmentation
Source: Sci Rep. 2020 Nov 23;10:20320. doi: 10.1038/s41598-020-76917-2 (PMC7683534; doi:10.1038/s41598-020-76917-2)
Supplement: Supplementary file 5 — Supplementary Information [file 41598_2020_76917_MOESM5_ESM.pdf]

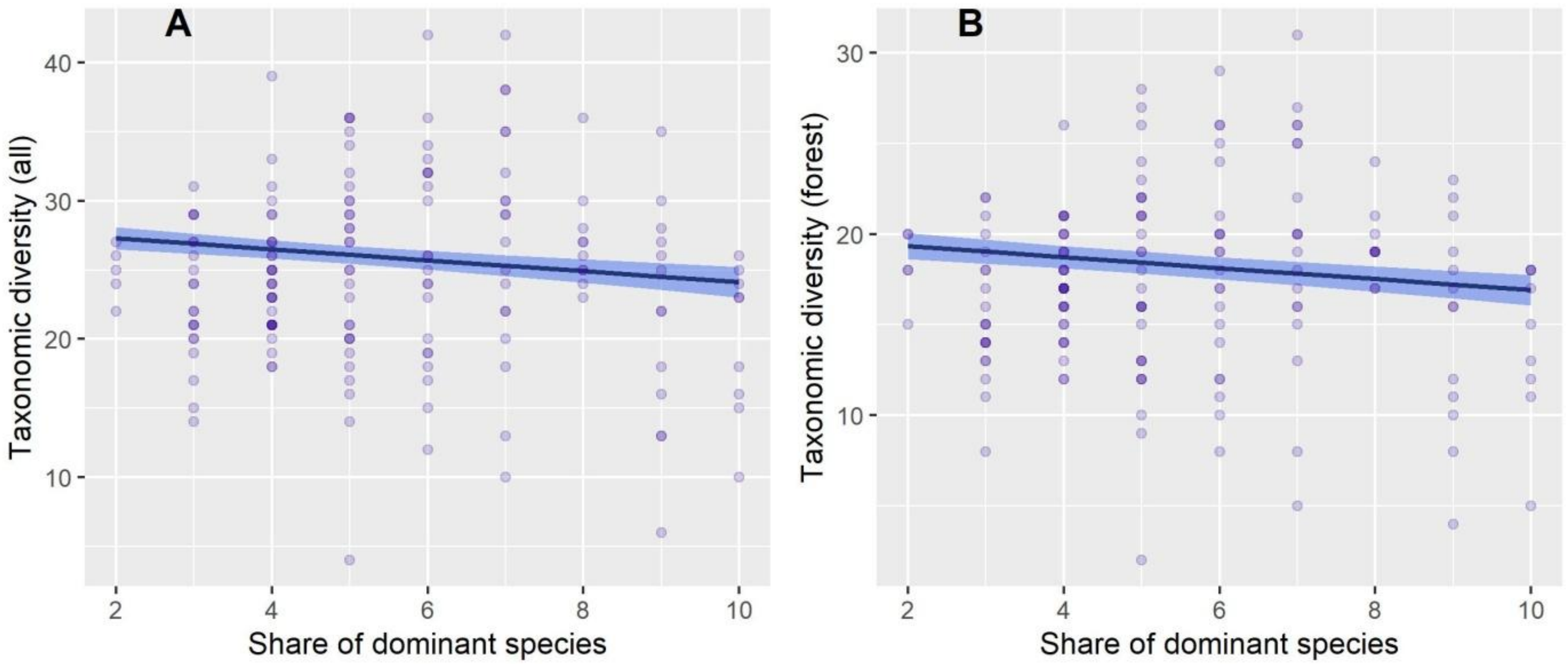

Figure S4. Response curves, derived from General Additive Modelling, showing the relationship between taxonomic diversity (calculated for all of the bird species and forest specialist group) and share of dominant species within a forest stand (ranging from 0 to 10).
